# Large-scale experiment to assess the collision impact force from a tsunami wave on a drifting castaway

**DOI:** 10.1371/journal.pone.0247436

**Published:** 2021-02-25

**Authors:** Tetsunori Inoue, Kazumasa Oguri, Hisami Suga, Kojiro Suzuki, Zdenek Prochazka, Takashi Nakamura, Akane Kurisu

**Affiliations:** 1 Marine Information and Tsunami Department, Port and Airport Research Institute, Yokosuka, Japan; 2 Department of Transdisciplinary Science and Engineering, Tokyo Institute of Technology, Yokohama, Kanagawa, Japan; 3 Research Center for Coastal Lagoon Environments, Shimane University, Matsue, Japan; 4 Research Institute for Global Change, Japan Agency for Marine-Earth Science and Technology, Yokosuka, Kanagawa, Japan; 5 Research Institute for Marine Resources Utilization, Japan Agency for Marine-Earth Science and Technology, Yokosuka, Kanagawa, Japan; 6 Coastal and Ocean Engineering Department, Port and Airport Research Institute, Yokosuka, Kanagawa, Japan; 7 Department of Information Engineering, National Institute of Technology, Oita College, Oita, Japan; 8 Akatutumi, Setagayaku, Tokyo, Japan; Al Mansour University College-Baghdad-Iraq, IRAQ

## Abstract

Although most fatalities in tsunami-related disasters are conjectured to be a result of drowning, injury risk owing to collision with other floating debris or fixed buildings has not been studied sufficiently. In this study, the impact force corresponding to the collision of a concrete block and drifting test body in a tsunami wave was experimentally investigated, and the injury risk was evaluated in terms of different biomechanical indexes; specifically, maximum acceleration, head injury criterion, and impact force. The injury risk indicated by the considered indexes was reasonably low. It was noted that if a healthy adult collided with a concrete wall under a velocity of 2.5 m s^-1^ and wave height of 0.59 m, the adult would likely not be critically injured. However, a similar collision impact poses considerable risk to infants and children, as well as the more sensitive regions of the adult body. Moreover, in the case of large tsunamis, such as that in the 2011 Great East Japan Earthquake, a drifting person may be at considerable risk for injuries. The collision impact occurring on the tip of a surge flow is notably significantly larger than that on a bore flow. This is because a surge flow, which arrives at the concrete block earlier than a bore flow, forms a certain water layer along the concrete wall and that layer acts as a cushion for any body drifting on the bore flow, indicating the importance of such a buffering effect. These findings can provide practical guidance regarding the formulation of effective tsunami-protection measures.

## Introduction

In tsunami-related disaster scenarios, most fatalities are conjectured to be a result of drowning; for instance, more than 90% of the fatalities arising from the tsunami that occurred owing to the Tohoku-Pacific Earthquake on March 11, 2011, were recorded as being due to drowning [1]. Consequently, existing studies concerning disasters on the waterside have primarily focused on drowning-related aspects. However, several of the deceased in such scenarios exhibit severe injuries [1], likely because of collisions with various debris and/or obstacles. Considering this aspect, Matsutomi [2] highlighted the injury risk owing to collisions with driftwoods in tsunami-related disaster scenarios. However, to date, the risk for a tsunami-drifted person owing to collisions with other debris and/or fixed obstacles has not been quantified.

Although the death or injury risk owing to collisions in traffic accidents or furniture overturning in earthquakes has been studied extensively [3,4], the literature related to the risk involved in the waterside in disasters is limited. Several researchers have examined the impact force of woody debris [5,6] or drifting cars [7] and ships [8] on a floodplain. Ikeno et al. [9] conducted large-scale open-channel experiments to examine the collision force of woody debris and its behaviour. Nistor et al. [10] effectively summarised these efforts. Nevertheless, whereas the damage to fixed structures such as buildings and piers has been evaluated in these studies, to date the damage to castaways has not been examined extensively.

Kurisu et al. [11] indicated that personal flotation devices (PFDs) are effective protection devices in tsunami events because a person wearing a PFD remains on the surface and is never dragged down into the water after being struck by tsunami waves. Nevertheless, the person may be injured owing to a collision with other floating debris or fixed buildings. Therefore, in this study, the impact force owing to the collision of a concrete block and a test body drifted by a tsunami wave was experimentally investigated based on acceleration data. The risk was evaluated considering three indexes, namely, the maximum acceleration, head injury criterion (HIC), and impact force, which are commonly used in the analysis of traffic accidents and turning-over and falling-down accidents. The findings are expected to provide practical guidance in the formulation of tsunami-protection measures.

## Materials and methods

### Experimental flume

**Fig 1:**
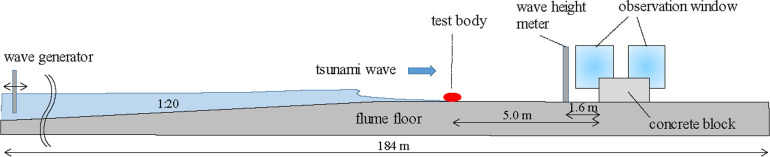
Schematic of the large hydro-geo flume.

The experiments were conducted using the Large Hydro-Geo Flume in the Port and Airport Research Institute, Yokosuka, Japan. The flume has length, width, and depth of 184 m, 3.5 m, and 12 m, respectively, with a piston-type wave-generator. The wave-generator is driven by a rack and pinion drive system including four AC servomotors with a maximum stroke of ±4 m, resulting in a maximum wave height of 3.5 m (Fig 1). Using this equipment, large-scale experiments can be conducted with model scales of 1/5 to 1/1 without any major scale effect problems [12].

A concrete block with length, width, and height of 1 m, 3 m, and 1 m, respectively, was installed on the flume bed [13] as the fixed object that strikes a test body. This concrete block was able to resist against the waves because of its heavy weight. In addition, a capacitance-type wave height meter (Denshi-Kogyo Co., Ltd., A3801) was installed 1.6 m windward from the concrete block.

### Test body

**Fig 2:**
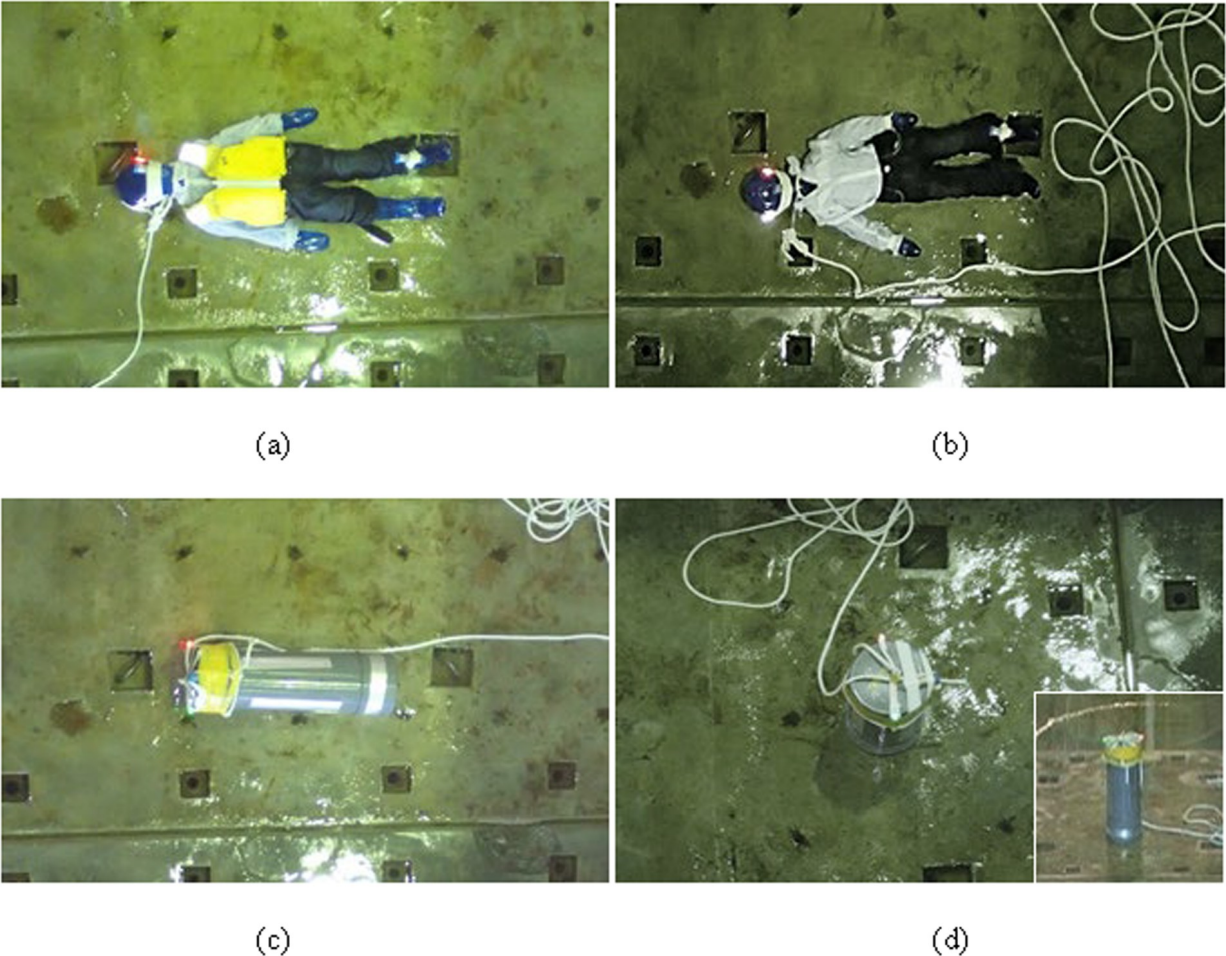
Test bodies used in the experiment (photos taken from a camera placed overhead): 3D kids model (KM) (a) with and (b) without PFD; PVC pipe in (c) lying position and (d) standing position (lower right shows the side view). The figures show the initial condition for each test body before being struck by a tsunami wave.

Several authorised dummies in the domain of car crash studies have been developed, such as Hybrid III, CRABI, and SID [14]. However, in this study, it was considered that the specific gravity of the test body should be approximately unity to reproduce the human body’s motion in a tsunami wave. Therefore, a soft polyvinyl chloride (PVC) dummy and a PVC pipe with lids filled with water were employed as test bodies. The PVC dummy was a 3D kids model (KM), which Nishida et al. [15] developed to support childsafe product design by using measurement data of 2,228 children aged 6 months to 13 y, in a collaborative work of human engineering researchers with product designers (Fig 2(A) and 2(B)). The model pertaining to a 6-year-old child in a standing posture was employed in this study. Although this model is usually filled with air (i.e., an air torso), it was filled with water to adjust the specific gravity to be approximately unity, resulting in a total weight of 20.0 kg. Following Kurisu et al. [11], one of the objectives of this study was to investigate the merits and demerits of wearing a PFD. Therefore, two configurations of the KM, that is, with and without PFD, were considered.

The second type of test body consisted of a simple PVC pipe with both ends sealed and filled with water. The height, diameter, and weight of the final pipe were 67.5 cm, 20.0 cm, and 25.4 kg, respectively (Fig 2(A) and 2(D)). The movement of the test bodies was recorded in the video sequence from the top and side (see below). To track the exact location of the bodies, green and red LED lamps were attached to the head of the KM and on top of the PVC pipe, respectively.

To investigate the impact of collisions, several researchers have used acceleration sensors to estimate the risk induced by the change rate of the velocity [16]. Therefore, in this work, two acceleration sensors (SysCom Corp., AccStick6) were attached to the left ear and left ankle of the KM, and one sensor of the same type was attached at the top of the PVC pipe. The acceleration data were collected at 400 Hz, which was the maximum rate within the acceptable range in our experiments.

### Experimental conditions

The experimental procedure was similar to that reported in [11]. The test body was initially situated on the flume floor 5.0 m windward from the front of the concrete block. In particular, KM lay in a supine position with an orientation of 90° to the wave direction. Although the PVC pipe was placed in the same orientation, the initial position was either standing or lying.

The test wave was generated with a paddle stroke of 9.0 m / 18 s, resulting in a solitary wave as a simulated-tsunami wave (maximum height of 0.59 ± 0.13 m at 1.6 m windward from the concrete block). The wave hit the test body, washed the body downstream, and stroked it in the front of the concrete block. The experimental conditions are summarised in Table 1.

**Table 1:** Experimental conditions. The time to collision refers to the time elapsed between the arrival of the tsunami wave to the test body and the collision of the test body and concrete block. The values were calculated considering the characteristic variations in the acceleration data. The acceleration data for the left ear of the KM in deployment No. 2 could not be recorded owing to device malfunction.

deployment No.	type of test body	sensor position	time to collision (s)	averaged drifting speed (m s-1)	maximum acceleration (G)
1	KIDS MODEL	left ear	1.85	2.70	8.88
		left ankle	1.90	2.64	7.35
2	KIDS MODEL	left ear	N.D.	N.D.	N.D.
		left ankle	1.79	2.79	9.26
3	KIDS MODEL	left ear	1.82	2.74	12.13
		left ankle	1.83	2.74	8.18
4	KIDS MODEL	left ear	1.80	2.77	11.48
		left ankle	1.75	2.85	10.86
5	PVC pipe	top	2.39	2.09	8.34
6	PVC pipe	top	2.29	2.19	4.46
7	PVC pipe	top	2.02	2.48	13.48
8	PVC pipe	top	2.07	2.41	12.70

The drifting of the test body by the tsunami waves was recorded at a frame rate of 60 frames per second by using a high-definition digital video camera (JVC Kenwood, GZ-V590) placed overhead, and the position of the test body was visually monitored. The drifting velocity was calculated with reference to the video image, distinctive variations in the acceleration data, and initial distance between test bodies and the concrete block. The exact time of collision was determined by referring to the video and time series of the acceleration data.

### Impact-force indexes

The impact force was evaluated considering the maximum acceleration, HIC, and average impact force. The HIC is generally used to determine the maximum tolerable severity or injury threshold in the case of traffic accidents and turning-over and falling-down accidents [17]. Specifically, HIC is one of the most widely referenced head injury assessment functions [17,18], representing the extent to which the brain is injured owing to a sudden acceleration. The index, which is defined as the time integral of linear acceleration, is especially valuable as it takes into account both the acceleration magnitude and duration [19].

In this study, HIC at the moment of collision was calculated from the time series of the acceleration data obtained from the sensors attached to the test body. It was determined as the maximum value of the product of the average acceleration to the power 2.5 and the time window width [3,17,20].

$$HIC{=\{{\left[\frac{1}{{(t}_{2}-t_{1})}∙\int_{t_{1}}^{t_{2}}\alpha (t).dt\right]}^{2.5}(t_{2}-t_{1})\}}_{max}$$ (1)

where, *α(t)* is the time series of acceleration, and *t*_*1*_ and *t*_*2*_ denote the start and end times of the time window, respectively. Because the time window width should ideally be less than 15 or 36 ms [21], its value was set as 15 ms, as recommended by the American Automobile Manufacturers Association [22]. According to [17], the condition *α*(*t*_1_) = *α*(*t*_2_) should be satisfied to maximise the right-hand side of Eq (1) and reduce the number of trials to find an appropriate time window.

The average impact force at the moment of collision was calculated using the following equation [26]:

$$\bar{F}=\frac{m\int_{t_{1}}^{t_{2}}\left(\alpha (t)dt\right)}{t_{2}-t_{1}}$$ (2)

where $\bar{F}$ is the average impact force, *α(t)* is the acceleration, and *m* is the weight of the test body. The same time window as in the case of HIC calculation was employed.

## Results

### General behaviour

**Fig 3:**
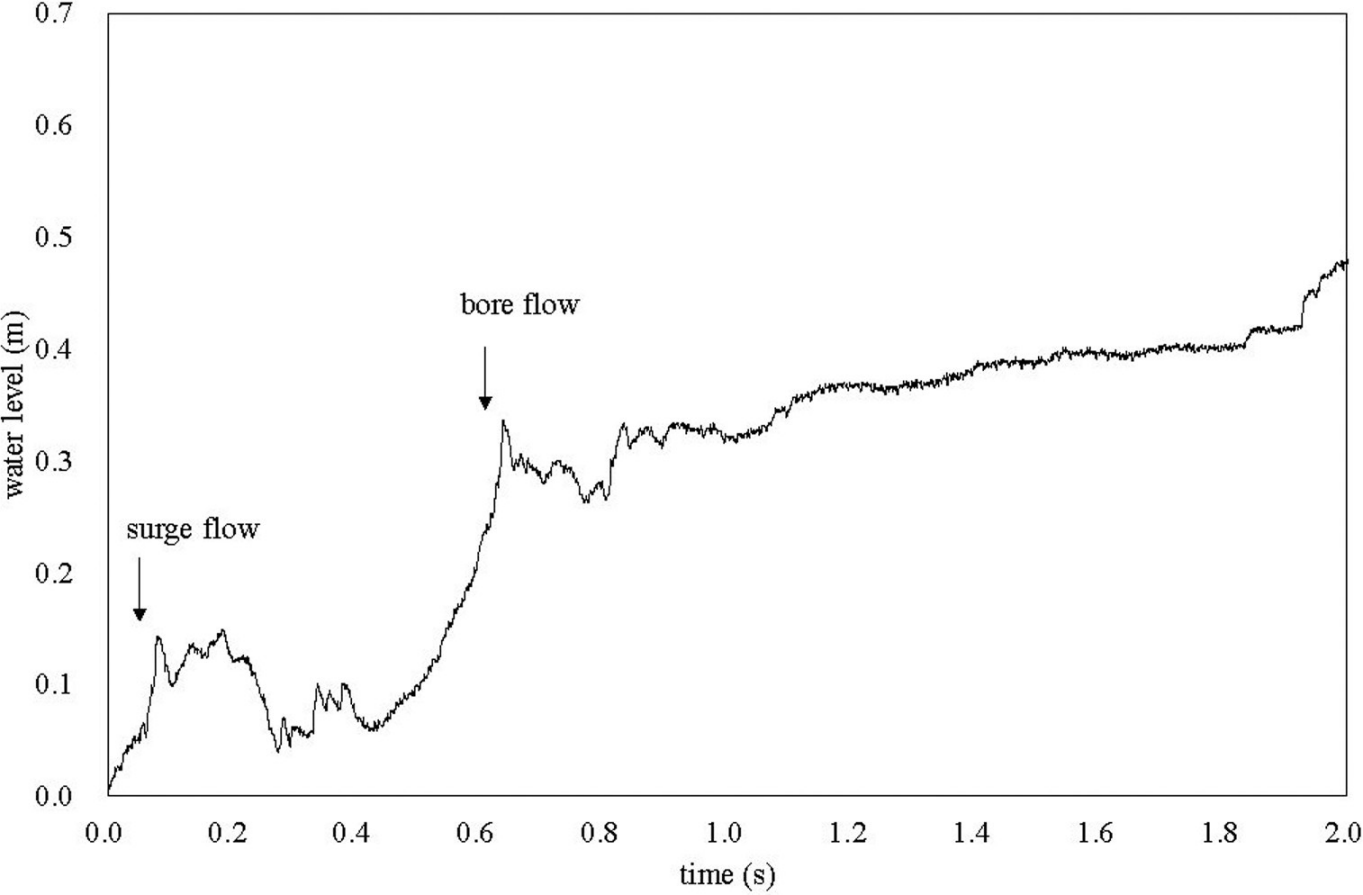
Time-course of water level measured 1.6 m from the concrete block. The horizontal axis shows the time elapsed since the wave reached the water level gauge.

First, the tip of the solitary wave generated using the paddle passed the front slope and reached the test body, while exhibiting a shallow flow. This shallow flow was followed by a deeper flow, owing to the wave generation condition and topography. Fig 3 shows the time-course of the water level measured 1.6 m windward from the concrete block in deployment 1. For the first 0.08 s after the wave arrival, the water level increased linearly to a value of approximately 0.12 m at 0.22 s, after which it decreased to 0.04–0.09 m. As the average flow velocity was 2.55 m s^-1^, the flow was considered to be a surge flow [23] that arrived antecedent to the larger water mass. After 0.42 s, the water level rapidly increased again and reached a value of 0.30 m, corresponding to a flow described as a bore flow [23]. Beyond this time, the water level slowly increased, exhibiting subcritical flow. The difference between surge and bore flows is discussed in the following text.

**Fig 4:**
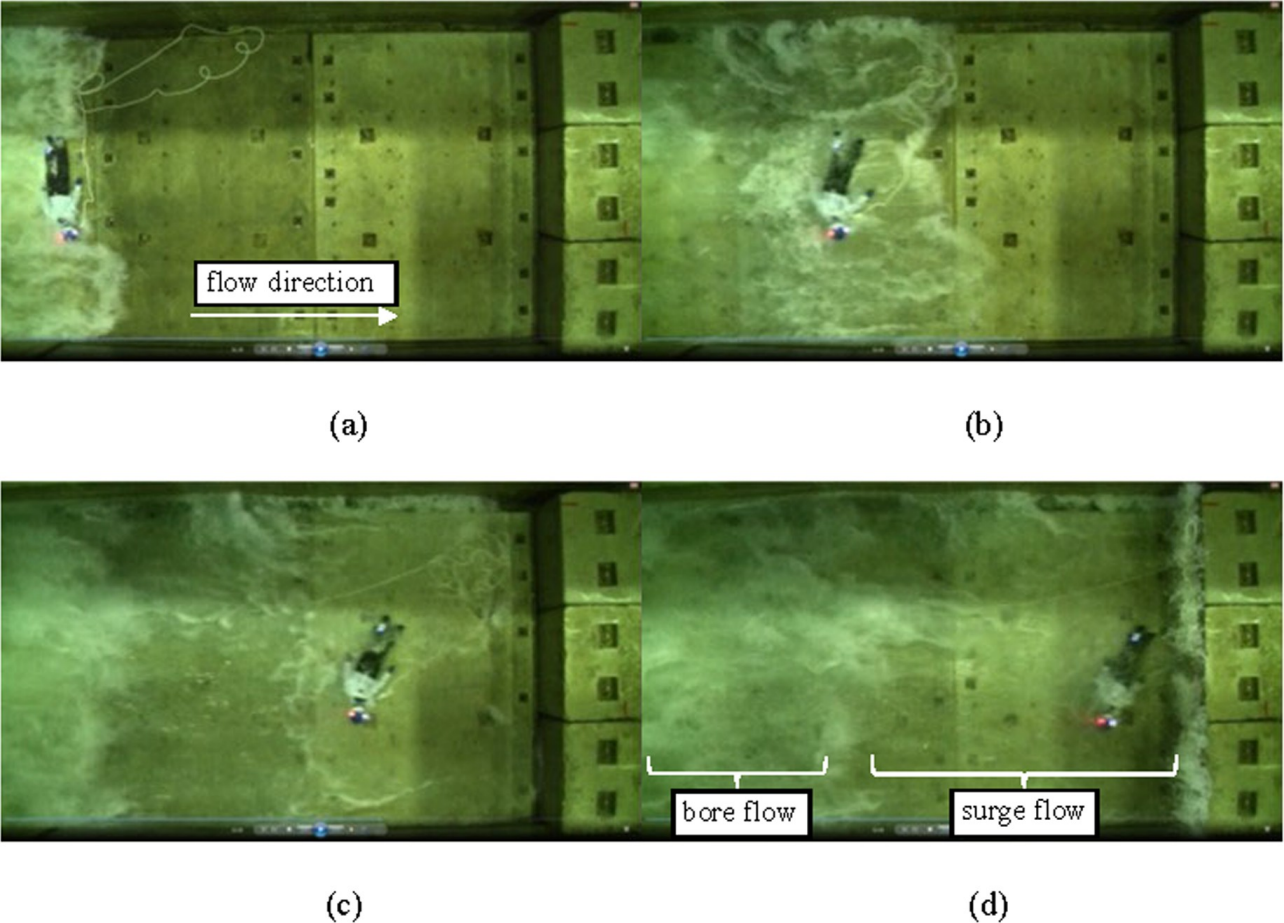
Frame recorded from the top view (KM, without PFD): (a) 0 s (b) ca. 0.4 s (c) ca. 1.5 s (d) ca. 1.8 s.

**Fig 5:**
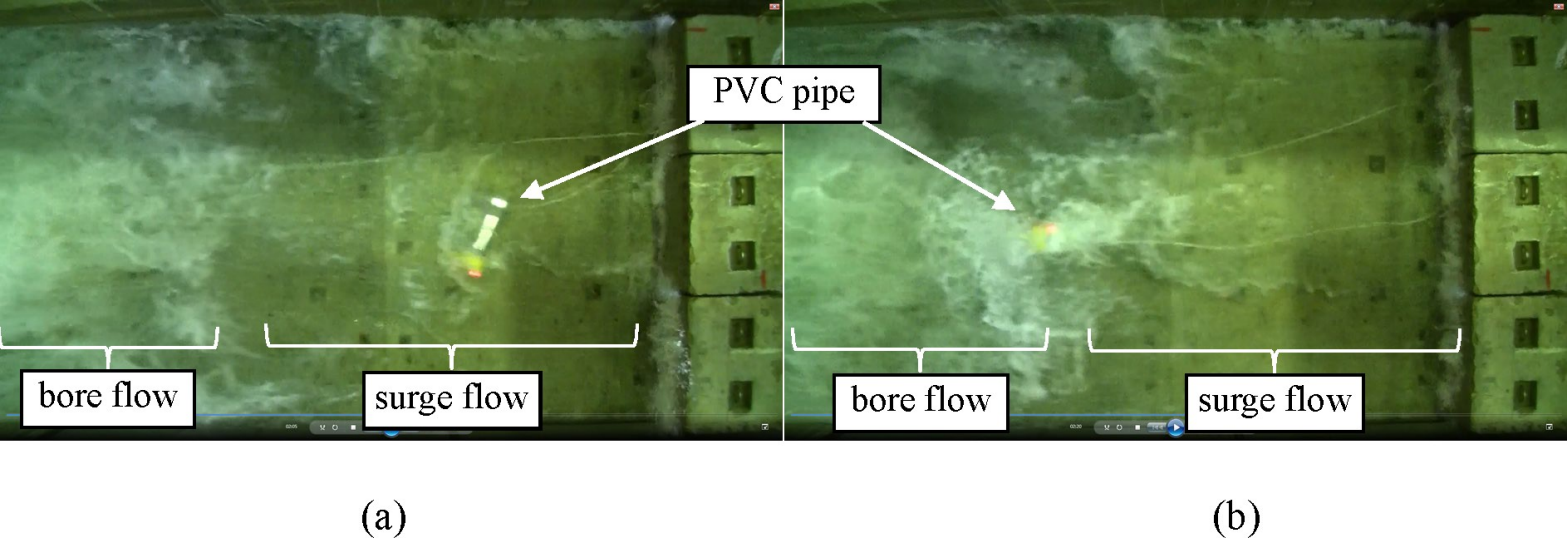
Frame recorded from the top view (PVC pipe): (a) initially lying (b) initially standing.

After being submerged by the waves, the KM and PVC pipe, which was initially in a lying position, were linearly transported to the concrete block (Fig 4(A)–4(D)), drifting on the surge flow immediately behind the tip of the wave (Figs 4(D) and 5(A)). The KM was transported while remaining in the supine position, whereas the PVC pipe was rolled over. The PVC pipe, which was initially in a standing position, retained the same posture and resisted the surge flow with slight skidding; nevertheless, the pipe was ultimately submerged and drifted by the bore flow (Fig 5(b)).

The time between the test body being submerged and the collision, average drifting speed, and average and maximum acceleration before and after the collision are listed in Table 1. The average travelling time to collision for the KM was 1.82 s and that for the initially standing and lying PVC was 2.34 s and 2.05 s, respectively. The difference in the values for the PVC pipe occurred because the standing PVC pipe required a certain duration to descend before it started drifting. Therefore, the average drifting velocity for the initially standing PVC pipe (2.14 m s^-1^) was smaller than that for the other bodies (2.44 m s^-1^).

### Acceleration, HIC, and impact force

**Fig 6:**
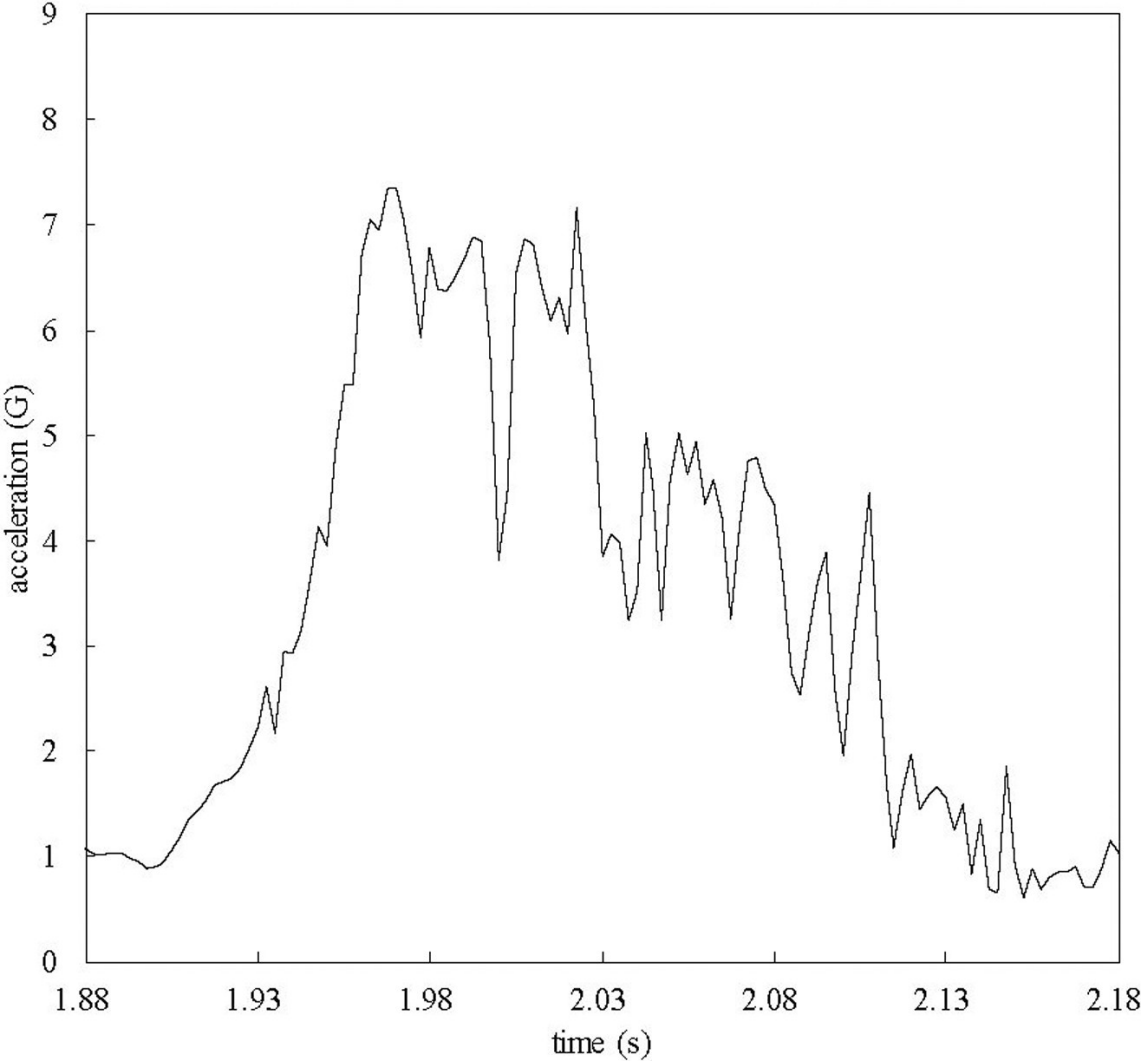
Time series of acceleration before and after the collision in deployment 1. The horizontal axis shows the time elapsed since the wave reached the test body.

An example time series of acceleration is shown in Fig 6. From these results, we decided that a sampling frequency of 400 Hz was sufficient to monitor the time series of acceleration. Owing to the collision, the acceleration exhibited a linear increase for the first 50–70 ms, followed by intense fluctuation for approximately 150 ms.

**Fig 7:**
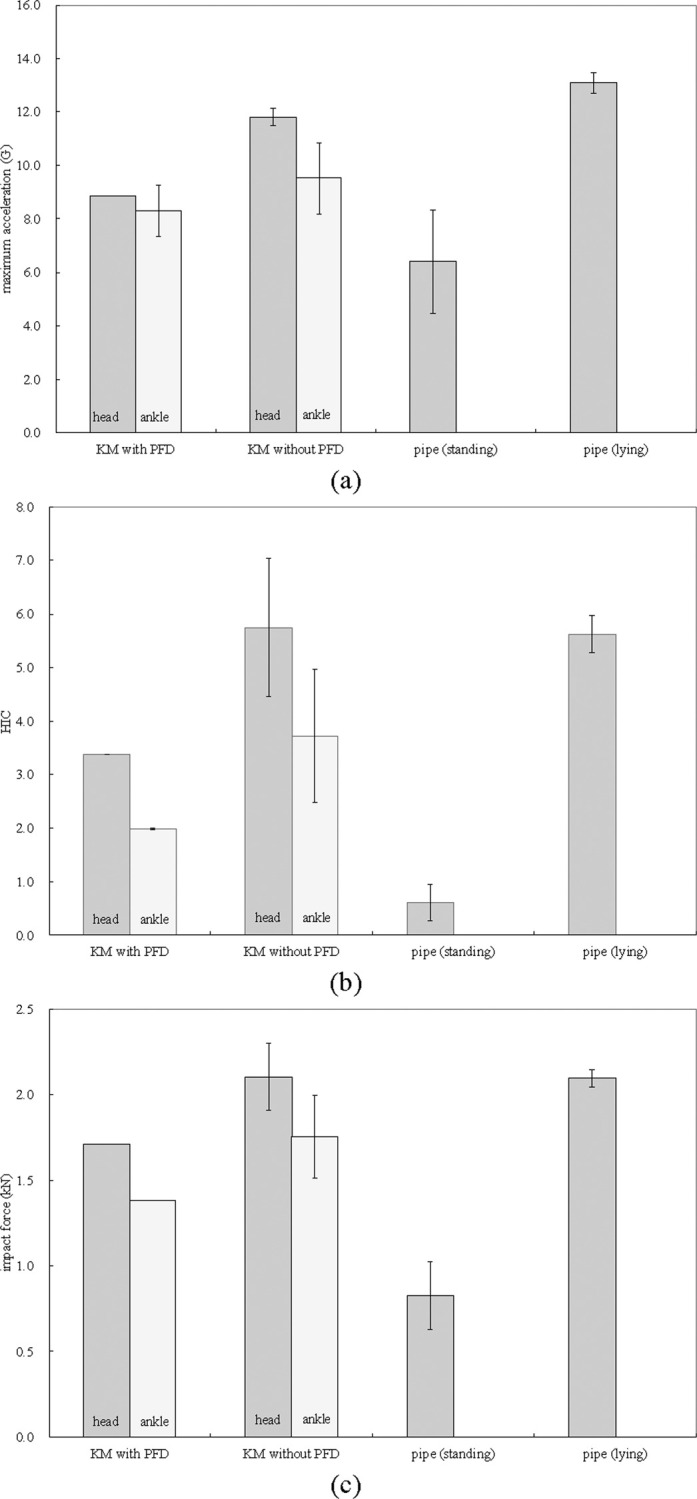
Impact-force indexes owing to collision: (a) Maximum acceleration (b) HIC (c) Average impact force.

The maximum acceleration, HIC, and average impact force exhibited the same qualitative tendency; specifically, the KM without the PFD and initially lying PVC pipe experienced a large impact, followed by that experienced by the KM with the PFD. The initially standing PVC pipe experienced the least impact (Fig 7).

Although the number of experiments is not sufficient to perform statistical analysis, the maximum acceleration value of the KM with PFD was smaller than that of the KM without PFD. The nearly equivalent time to collision and average drifting velocity indicate that the life jacket can absorb a certain amount of shock.

The difference in the values pertaining to the initially standing (deployments 5 and 6) and lying (deployments 7 and 8) PVC pipes is substantial owing to the specific behaviour of the PVC pipe after being submerged by the wave. The initially standing PVC pipe maintained a standing posture for the first 0.5 s and resisted being submerged by the wave, whereas the initially lying PVC pipe started rolling at the same time as the wave impacted. Owing to this momentary persistence of the standing PVC pipe in the initial stage, the average drifting velocity and maximum acceleration slightly and substantially decreased, respectively. This finding can provide a basis to develop effective protection measures against tsunami waves, as discussed in the subsequent section.

## Discussion

### Evaluation of collision impact

The considered experimental condition involving a flow velocity of 2.5 m s^-1^ falls within the tsunami conditions with a velocity range of 2 to 5 m s^-1^, as reported in [24]. Therefore, the obtained results can be considered to be practically relevant in actual tsunami disasters. It is worth noting that, although a KM and PVC pipe bearing a child’s weight were employed in this experiment, the discussion below can be extended to adults as well. In this regard, the density and inertia of a test body are important in understanding its behaviour in a tsunami wave. Concerning the density, the test bodies employed here were filled with water to achieve neutral buoyancy. As for the inertia, because the drifting force of a tsunami wave was significantly large relative to the test bodies, it is safe to assume that the difference in weight will not alter the behaviour of test bodies. This was qualitatively confirmed from the preceding study employing an adult test body [11].

The maximum acceleration values for KM and PVC pipe were 12.13 G and 13.48 G, respectively, which correspond to a low risk for severe injury [14]. In terms of the head injury risk, the injury assessment reference values, which are biomechanically valid criteria for assessing the likelihood of injury [25], for HIC_15_ and HIC_36_ were 700 [14] and 1000 [3], respectively. Regardless of the presence of a PFD, the HIC values (HIC_15_ = 0.28–7.04) were considered to be relatively low in terms of posing an injury risk [14]. In addition, the average impact force calculated using Eq (2) ranged from 0.63 to 2.30 kN. Ikuta et al. [26] reported that for an average impact force of 5.0 kN, the probability of a femur fracture was 0.2%, which is considerably low. While these calculations were conducted using the values 20.0 and 25.4 kg for m in Eq (2), using higher weights (representative of adults’ weight) will not affect the overall conclusion for the impact force aspect. Considering the values of acceleration, HIC, and average impact force, it can be concluded that a collision with a concrete wall under the given conditions is not likely to cause critical head injuries or fracturing of strong parts, such as femurs, of a healthy adult body. These results can be considered reasonable because the drifting velocity of approximately 2.5 m s^-1^, i.e., approximately 9.3 km h^-1^, is considerably smaller than that in a typical major traffic accident.

Nevertheless, several authors have reported considerably low values as criteria for injury occurrence. Ono [27] stated that facial bones are generally weak, and their tolerance strength ranges from 0.6–3.0 kN. In this regard, the values of the average impact force obtained in this study (0.63–2.30 kN) may indicate a potential risk for the occurrence of fractures in the facial area. The injury assessment reference values for shoulder, abdomen, and tibia fractures for 6–10 y old children, according to [14], are comparable with the values obtained in this study. Moreover, according to [21], when scaling for people of different sizes and age groups, the geometric differences do not fully account for the differences in the tolerance to loading. Moreover, gender [16] and health status [28] are also critical properties for the injury risk. These aspects must be examined in a future work. In particular, the collision owing to a tsunami wave likely poses a potential risk for infants, children, and more sensitive regions of the human body.

Moreover, Ohta and Yamanaka [29] reported that the drifting velocity of certain objects submerged by the tsunami wave in the 2011 Great East Japan Earthquake ranged from 2.3 to 16.4 m s^-1^. The maximum value is approximately 6.4 times larger than the mean drifting velocity measured in the experiments in this work. Assuming that the acceleration owing to a collision is directly proportional to the drifting velocity, the average impact force and HIC are expected to be approximately 40 and 100 times larger than the estimated values in this study, respectively. This finding indicates that the average impact force and HIC may exceed the critical values in the case of a large tsunami.

### Difference in impact force between surge and bore flows

In this section, the differences in HIC and impact force pertaining to the initially standing (deployments 5 and 6) and lying (deployments 7 and 8) PVC pipes are discussed. The average drifting velocities of the initially standing and lying PVC pipes were 2.14 m s^-1^ and 2.44 m s^-1^, respectively. Because HIC is proportional to the 2.5th power (Eq (1)) of the time-integrated acceleration (i.e., velocity), the ratio of HICs of the initially standing and lying PVC pipes was considered to follow the 2.5th power of the velocity ratio, 0.72 (= 2.14^2.5^/2.44^2.5^). However, the average values of HICs, calculated by Eq (1), of the initially standing and lying PVC pipes were 0.61 and 5.62, respectively, and their ratio was 0.11 (= 0.61/5.62). Similarly, the average impact force values of the initially standing and lying PVC pipes were 0.83 kN and 2.10 kN, respectively, and their ratio was 0.40 (= 0.83/2.10). Although the average impact force is proportional (Eq (2)) to the velocity, the ratio of 0.40 is considerably smaller than the velocity ratio (e.g. 0.88 = 2.14/2.44). Therefore, the differences in HIC and average impact force pertaining to the initially standing and lying PVC pipes cannot be attributed to the difference in the drifting velocities.

**Fig 8:**
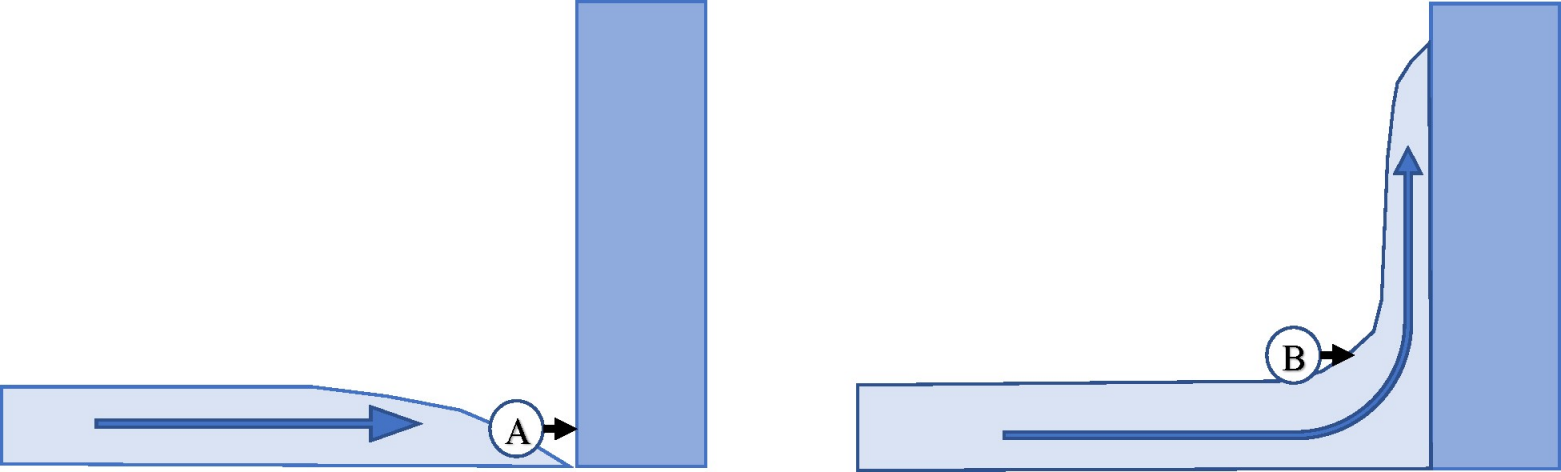
Schematic of the collision of the PVC pipe on the (A) surge flow and (B) bore flow. The PVC pipe in the surge flow directly hits the wall and experiences a certain impact. In contrast, the PVC pipe in the bore flow hits the wall covered with a water layer and thus experiences a lower impact owing to the buffering effect.

As mentioned previously, the initially lying PVC pipe was submerged and transported by the surge flow. In contrast, the initially standing PVC pipe resisted the flow for approximately 0.5 s and was finally submerged by the bore flow. Consequently, the initially lying PVC pipe directly impacted the wall, whereas the initially standing pipe impacted the wall covered by a water layer owing to the splashing of the surge flow (Fig 8). Matsutomi [2] conducted experiments in a mesoscale channel to estimate the impulse force on structures owing to a collision with driftwoods on a tsunami wave. The author mentioned that the buffering effect of the water covering a structure [30] helped to reduce the impact force of the collision. In this study as well, the buffering effect likely reduced the average impact force and HIC values of the initially standing PVC pipe.

This result indicates that to avoid injury risks owing to collisions in flowing water, such as in tsunami evacuation scenarios, the buffering effect of water can be exploited. In particular, to reduce the collision impact, it is desirable to resist the first impact due to the surge flow and delay being submerged by the wave.

### Other aspects

The KM was made with soft PVC and filled with water (i.e., water torso). Therefore, its physical properties (such as the stiffness) were likely different from those of the human body and influenced the impact-force calculation results. Nevertheless, the PVC pipe used in this work, which had a ‘hard skin’, exhibited similar impact force and HIC values as those of the KM. Therefore, it was considered that the material of the KM did not notably influence the impact force in this study.

Moreover, in this work, the test bodies were drifted by a water flow with a depth less than the concrete block height, and they inevitably impacted the wall. In the case of water with a large depth, the water likely flows over a structure, and the risk of collision of a floating person with a structure is lower. In this context, PFDs can be used to effectively avoid collisions with hard structures.

### Usability of acceleration sensor

Because acceleration has been observed to be a severity parameter that best explains the risk of injury and crashing [17], acceleration sensors have been widely applied in the research fields of traffic accidents [3], sports medicine [20], and civil engineering [10]. In the analysis process, the collision impact is simply considered to be in proportion to the acceleration to the power of 2.5 [3,4]. In this regard, the acceleration sensor is a useful and practical analysis tool that can be used even by novice operators.

Although the magnitude of the acceleration value was measured and analysed in this study, the acceleration has three rotational degrees of freedom. Moreover, a rotational acceleration sensor can provide information regarding different aspects, such as the behaviour of a human body drawn into a vortex and an estimate of the diffuse axonal injury. Newman et al. [18] highlighted that the rotational acceleration, which is not taken into account in this study, plays a significant role in the occurrence of a mild traumatic brain injury. Therefore, more effective and active use of an acceleration sensor must be realised in future work.

## Conclusion

Because drowning is conjectured to be the main cause of death during a tsunami, a key protection measure is to remain on the water surface. Considering this aspect, in a previous work, the authors demonstrated that the use of PFDs is effective to prevent drowning during a tsunami [11]. In this regard, the objective of this study was to examine the injury risk for a drifting person in a tsunami wave. The experimental conditions, pertaining to a flow velocity of 2.5 m s^-1^ and water depth of less than 0.5 m, corresponded to low injury risk for healthy adults that may collide with a concrete block. However, the calculated impact force and HIC values were comparable with certain tolerance criteria, especially for infants and children and more sensitive parts of the adult human body. In a large tsunami disaster, the drifting velocity is expected to be considerably higher than that in the experimental conditions, thereby corresponding to a higher risk. Moreover, in an actual tsunami disaster, the victims may also be exposed to a risk of colliding with other drifting debris.

It was observed that the collision impact owing to a bore flow was considerably lower than that owing to a surge flow. This phenomenon occurs because of the buffering effect of the water that reaches the concrete block in advance, thereby indicating that in tsunami evacuation scenarios, it is desirable to resist the first impact owing to the surge flow and delay being submerged.

As the number of experiments was insufficient to perform a valid statistical analysis, it is necessary to validate the results by conducting more experiments. Moreover, as the impact force and human damage are site-specific and cannot be evaluated from a simple theory, interdisciplinary and/or statistical knowledge should be gathered through various laboratory and field experiments considering not only the physical aspects but also biomechanics. Numerical simulations pertaining to a human body in water [31] can be a valuable tool for further examining these aspects.
